# Genetic characterization at the species and symbiovar level of indigenous rhizobial isolates nodulating *Phaseolus vulgaris* in Greece

**DOI:** 10.1038/s41598-021-88051-8

**Published:** 2021-04-21

**Authors:** Evdoxia Efstathiadou, Georgia Ntatsi, Dimitrios Savvas, Anastasia P. Tampakaki

**Affiliations:** 1 Laboratory of General and Agricultural Microbiology, Department of Crop Science, Agricultural University of Athens, Iera Odos 75, Votanikos, 11855 Athens, Greece; 2 Laboratory of Vegetable Production, Department of Crop Science, Agricultural University of Athens, Iera Odos 75, Votanikos, 11855 Athens, Greece

**Keywords:** Microbiology, Bacteria, Symbiosis

## Abstract

*Phaseolus vulgaris* (L.), commonly known as bean or common bean, is considered a promiscuous legume host since it forms nodules with diverse rhizobial species and symbiovars. Most of the common bean nodulating rhizobia are mainly affiliated to the genus *Rhizobium,* though strains belonging to *Ensifer, Pararhizobium, Mesorhizobium, Bradyrhizobium,* and *Burkholderia* have also been reported. This is the first report on the characterization of bean-nodulating rhizobia at the species and symbiovar level in Greece. The goals of this research were to isolate and characterize rhizobia nodulating local common bean genotypes grown in five different edaphoclimatic regions of Greece with no rhizobial inoculation history. The genetic diversity of the rhizobial isolates was assessed by BOX-PCR and the phylogenetic affiliation was assessed by multilocus sequence analysis (MLSA) of housekeeping and symbiosis-related genes. A total of fifty fast-growing rhizobial strains were isolated and representative isolates with distinct BOX-PCR fingerpriniting patterns were subjected to phylogenetic analysis. The strains were closely related to *R. anhuiense*, *R. azibense*, *R. hidalgonense, R. sophoriradicis*, and to a putative new genospecies which is provisionally named as *Rhizobium* sp. I. Most strains belonged to symbiovar phaseoli carrying the α-, γ-a and γ-b alleles of *nodC* gene, while some of them belonged to symbiovar gallicum. To the best of our knowledge, it is the first time that strains assigned to *R. sophoriradicis* and harbored the γ-b allele were found in European soils. All strains were able to re-nodulate their original host, indicating that they are true microsymbionts of common bean.

## Introduction

*Phaseolus vulgaris* (L.), commonly known as bean or common bean, is an important legume crop that is cultivated worldwide as a grain or vegetable crop in many parts of the tropics, subtropics, and temperate regions**.** In southern Europe, the main common bean producers are Greece, Italy, and Spain (FAOSTAT, 2019) which highlights the socioeconomic importance of this legume crop. In Greece, the main growing areas of bean production are located in north and center of the country accounting for 40.4% of pulse arable land with an annual production of approximately 79,340 tons (FAOSTAT, 2019). Apart from this, traditional farmers still cultivate their own landraces contributing to the national bean production. However, the cultivation of common bean requires high amounts of nitrogen fertilizers which increase production costs and contribute to increased environmental impacts. One sustainable approach to diminish the use of N fertilizers is the exploitation of the Biological Nitrogen Fixation (BNF) that provides nitrogen to plants, in the form of ammonia, through the symbiotic association of legumes with rhizobia.

*P. vulgaris* establishes symbiotic associations, forming nitrogen-fixing root nodules, with diverse rhizobia in different countries and continents^1,2^. Common bean is very promiscuous in its association with rhizobia since it is nodulated by genetically diverse rhizobial species, which are mainly affiliated to the genus *Rhizobium,* though strains belonging to *Ensifer*, *Pararhizobium*, *Mesorhizobium*, *Bradyrhizobium*, and *Burkholderia* have also been reported. To date, more than thirty rhizobial species have been found to form symbiotic associations with common bean such as *R. aethiopicum*^3,4^*, R. acidisoli*^5^, *R. anhuiense *^6^, *R. azibense*^7^, *R. ecuadorense*^8^, *R. esperanzae*^9^, *R. etli*^10^, *R. freirei*^11^, *R. gallicum*^12^, *R. giardinii*^12^, *R. hidalgonense*^13^, *R. leguminosarum*^10^, *R. leucaenae*^14^, *R. lusitanum*^15^, *R. mesoamericanum*^16^, *R. mongolense*^17^, *R. paranaense*^18^, *R. phaseoli*^19^, *R. sophorae* and *R. sophoriradicis*^20^, *R. tibeticum*^21^, *R. tropici*^22^, *R. vallis*^23^, *E. meliloti*^24^, *E. fredii*^25^ *E. medicae*^26^, *E. americanum*^27^, *Mesorhizobium* sp.^28^, and *Bradyrhizobium* sp.^29^. Rhizobial species belonging to Betaproteobacteria, such as *Burkholderia phymatum* was also found capable of forming nodules on common bean plants^30^. Moreover, several bean-nodulating strains either misnamed or with uncertain species affiliation were recently assigned to validly described species or to novel *Rhizobium* lineages based on genomic data^6^.

The promiscuity of common bean is not only related to the rhizobial species but also to the symbiovar. Up to date, eight symbiovars (phaseoli, mimosae, gallicum, orientale, giardini, tropici, mediterranense, unnamed) distributed in diverse rhizobial species have been found in common bean nodules^2,31,32^. All symbiovars, except of mediterranense, are linked to the genus *Rhizobium*, while the symbiovars giardini, gallicum, and phaseoli are also linked to the genus *Pararhizobium*^33^*.* The sv. mediterranense is linked to the genus *Ensifer* and an unnamed symbiovar has been found in *R. grahamii* and *R. mesoamericanum*^16,31^. Among the various symbiovars found in bean nodulating rhizobia, the sv. phaseoli is the most prevalent worldwide and distributed in various chromosomal backgrounds such as *R. phaseoli*, *R. leguminosarum*, *R. etli*, *R. sophoriradicis*, *R. vallis*, *R. giardini*, *R. gallicum*, *R. lusitanum*, *R. ecuadorense, R. sophorae*^8,10,12,15,19,20,23^. Noteworthy, the sv. phaseoli has a narrow host range, limited to *P. vulgaris* while the other symbiovars have a broader host range^12,31,34^. For instance, the sv. tropici found in *R. tropici*, *R. leucaenae*, *R. lusitanum*, and *R. freirei* can nodulate, apart from *P. vulgaris,* several other legumes such as *Leucaena leucocephala and Macroptilium atropurpureum*^11,14,15,35^. Besides, the sv. mediterranense harbored by *E. meliloti*, *E. fredii,* and *E. americanum* confers nodulation and nitrogen fixation on *P. vulgaris*, *L. leucocephala,* and *Acacia*^24,36^^.^ The sv. mimosae has also a broad host range nodulating *Mimosa affinis*, *L. leucocephala* as well as *P. vulgaris*^37^.

Many studies on rhizobia nodulating *Phaseolus vulgaris* have revealed that *R. etli* and *R. phaseoli* of the sv. phaseoli are the predominant bean nodulating rhizobia in both the Mesoamerican and Andean centers of origin, though strains belonging to other rhizobial species, such as *R. tropici*, *R. leguminosarum*, *R. gallicum,* have also been reported^10,22,38–43^. Most of the American rhizobial species nodulating common bean have also been found in other continents indicative of their American origin and distribution with bean seeds^44,45^. However, many other rhizobial species have also been isolated from bean nodules in Europe, Africa, and Asia, where common bean has been introduced later. This suggests that resident rhizobia of the introduced regions might obtain symbiotic genes by horizontal transfer from the American strains. In support of this, several European and African strains (with identical or different chromosomal backgrounds) share similar symbiotic genes to those found in American strains^12,15,46,47^.

In European soils, the sv. phaseoli, gallicum, tropici, giardinii and mediterranense have been found in diverse rhizobial species such as *R. leguminosarum*, *R. etli*, *R. tropici*, *R. gallicum*, *R. lusitanum*, *R. giardinii*, *E. fredii*, and *E. melilot*i. In particular, strains of sv. phaseoli have been found in Spain^45–48^, France^49^, England^50^, and Austria^51^ and the sv. gallicum in Austria^52^ and France^12^. The sv. tropici of *R. lusitanum* was found in Portugal^15^, while the sv. giardinii and mediterranense in France and Spain, respectively^12^.

Despite that common bean can establish symbiotic relationships with a great number of rhizobial species carrying different symbiovars, it displays reduced BNF ability compared to other legumes and thus it is considered a poor nitrogen fixer pulse^45,53–57^. For this reason, the selection of suitable varieties or landraces of common bean with high nitrogen fixation capacity in combination with efficient, competitive, and well-adapted rhizobial strains in different edaphoclimatic zones is considered the most sustainable agricultural practice for maximizing nodulation and nitrogen fixation in common bean and finally achieving optimal biofertilization.

Knowledge about the diversity of rhizobia nodulating common bean in Greece is very limited. Recently, a study analysed the bean rhizobial population in a geographically isolated region, Prespa lakes plain, located in the Northern Greece^58^. Although the isolates were not identified at the species and symbiovar level, analysis of the 16S-23S internal transcribed spacer region showed that they were related to *R. leguminosarum*, *R. etli*, *R. gallicum, R. mongolense*, and *E. meliloti*.

The aim of the present study was to isolate and characterize rhizobia that nodulate local common bean varieties grown in five different edaphoclimatic regions of the mainland and the islands of Greece that have not previously been analysed. The genetic diversity of the isolates was assessed by DNA fingerprinting analysis and their phylogenetic affiliation at the species level was determined by sequencing analyses of 16S rRNA, *recA*, *atpD*, *glnII,* and *gyrB*. The taxonomic position at the symbiovar level was determined by analyses of the widely used symbiosis genes *nifH* and *nodC*.

## Results and discussion

### BOX-fingerprinting

A total of 50 rhizobial strains were isolated from nodules of local common bean varieties grown in five different geographical regions located in the northern mainland of Greece (Imathia, Metsovo, Preveza) as well as in the Greek islands Karpathos, and Tinos (Supplementary Fig. S1). Strains were named “PV”, representing the host *P. vulgaris* (PV) followed by two letters representing the region of isolation. Strains isolated from Imathia, Metsovo, Preveza, Karpathos, and Tinos were named either “IM” or “MT” or “PR” or “KA” or “TN”, respectively. All isolates were fast-growing, acid-producing bacteria that formed effective pink–red coloured nodules (Nod+/Fix+) on their host of origin (Table 1).

**Table 1:** Characteristics of rhizobial strains obtained in this study and their phylogenetic relationships with the closest type species.

Strain^a^	BOXtype^b^	No isolates^c^	MLSA Clade	16S^d^	MLSA^d^	Strain definition	Symbiovar	*nodC* allele	Nodulation^e^	Geographic origin
PVKA6	1	7	1	Rph ATCC 14482^T^ (99.85%)	Rso CCBAU 03470^T^ (95.21%)	New lineage	Phaseoli	α	NodC+/Fix+	Karpathos
PVIM10	1	-	1	Rph ATCC 14482^T^ (99.85%)	Rso CCBAU 03470^T^ (95.21%)	New lineage	Phaseoli	α	NodC+/Fix+	Imathia
PVMT25	2	1	1	Rph ATCC 14482^T^ (99.85%)	Rso CCBAU 03470^T^ (95.26%)	New lineage	Phaseoli	α	NodC+/Fix+	Metsovo
PVTN21	3	23	2	Rso CCBAU 03470^ T^ (99.92%)	Rso CCBAU 03470^T^ (100%)	*R. sophoriradicis*	Phaseoli	γ-b	NodC+/Fix+	Tinos
PVPR1	4	5	3	Ran CCBAU 23252^T^ (100%)	Ran CCBAU 23252^T^ (99.44%)	*R. anhuiense*	Phaseoli	γ-a	NodC+/Fix+	Preveza
PVMT26	5	7	4	Ran CCBAU 23252^T^ (100%)	Rhi FH14^T^ (99.59%)	*R. hidalgonense*	Phaseoli	α	NodC+/Fix+	Metsovo
PVIM1	6	7	5	Rya SH22623^T^ (99.92%)	Raz 23C2^T^ (99.75%)	*R. azibense*	Gallicum	N/A	NodC+/Fix+	Imathia

Ran, *Rhizobium anhuiense*; Raz, *Rhizobium azibense*, Rhi, *Rhizobium hidalgonense*; Rph, *Rhizobium phaseoli*; Rso, *Rhizobium sophoriradicis*; Rya, *Rhizobium yanglingense*; *N/A* not applicable.

^a^Representative isolates from different BOX-groups and geographic regions.

^b^Different numbers were assigned to represent each BOX-PCR pattern.

^c^Number of isolates displaying identical BOX-PCR pattern.

^d^Percent identities determined by multiple sequence alignments of partial gene sequences using the algorithm CLUSTAL Omega at https://www.ebi.ac.uk/Tools/msa/clustalo/.

^e^Nodulation was tested on common bean, the host of origin for each isolate.

The genetic diversity of the rhizobial isolates was firstly analyzed by BOX-PCR fingerprinting, which allows the differentiation among strains even of the same rhizobial species^59^. The isolates displayed six distinct BOX-PCR profiles (Table 1, Supplementary Fig. S2). The isolates in each BOX profile shared identical fingerprints indicating that they might be clones. Noteworthy, isolates obtained from plants at different sampling sites displayed different BOX profiles, except for one, represented by PVKA6, which was present in isolates from Imathia, Karpathos, and Metsovo (Supplementary Fig. S2). Representative strains of each profile were chosen for further phylogenetic analysis.

### 16S rRNA gene analysis

**Figure 1:**
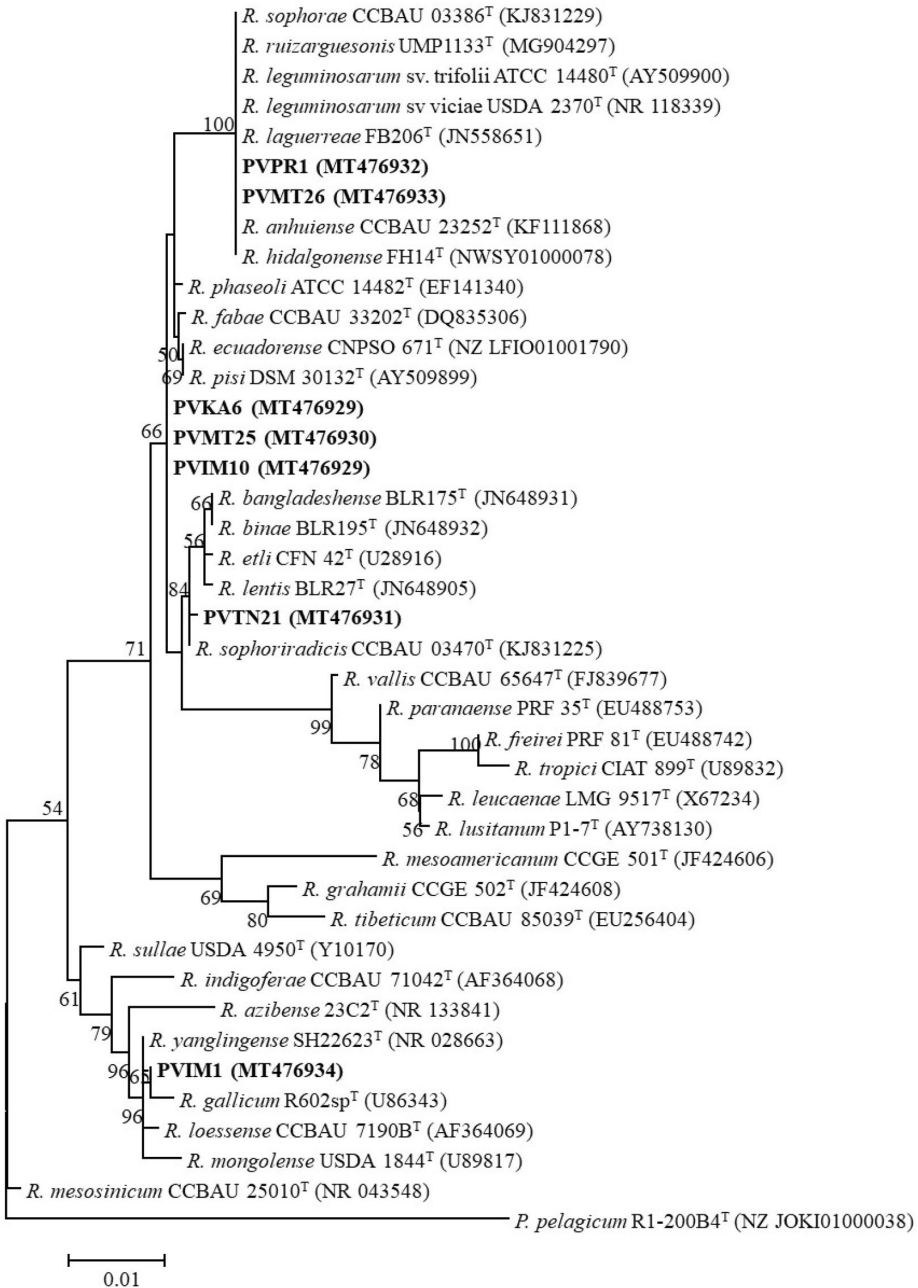
Maximum likelihood phylogenetic tree based on nearly complete 16S rRNA gene sequences (1308 bp) showing taxonomic relationships of the strains representing the different BOX groups. Strains isolated in the present study are shown in boldface and their accession numbers are given in Supplementary Table S2. Type strains are indicated by superscript “T” and the GenBank accession numbers of the *rrs* sequences are indicated within parentheses. Bootstrap values (greater than 50%) were calculated for 500 replications and are shown at the nodes. The scale bar shows the number of nucleotide substitutions per site. Phylogenetic analysis was conducted in MEGA 6^104^ (https://www.megasoftware.net/) using the maximum likelihood algorithm with the Kimura 2 parameter model plus Gamma rate distribution plus invariant site (K2 + G + I). *Pseudorhizobium pelagicum* R1-200B4^T^ was used as outgroup to root the tree. The genus names are abbreviated as follows: *R.*, *Rhizobium*; *P.*, *Pseudorhizobium*.

According to the BOX grouping results, seven isolates (PVIM1, PVIM10, PVKA6, PVMT25, PVMT26, PVPR1, and PVTN21) representing six different BOX patterns and originating from different geographic regions were chosen for subsequent analyses. Nearly full-length *rrs* gene sequences (> 1350 bp) were determined for all representative isolates and a region of 1308 bp was considered for the alignment. The 16S rRNA gene phylogenetic tree showed that all isolates were closely related to the defined species within the genus *Rhizobium* (Fig. 1).

The strains PVKA6, PVIM10, and PVMT25 displayed identical *rrs* sequences and were clustered in a distinct group which was closely related to the type strains of *R. phaseoli* ATCC 14482^T^, *R. fabae* CCBAU 33202^T^, *R. ecuadorense* CNPSO671^T^, and *R. pisi* DSM 30132^T^ with a 99.85% identity. The *rrs* sequence of PVTN21 was clustered along with *R. sophoriradicis* CCBAU 03470^T^ with 99.92% identity. The strains PVMT26, and PVPR1 were clustered on a well-supported branch containing *R. anhuiense* CCBAU 23252^T^, *R. hidalgonense* FH14^T^, *R. laguerreae* FB206^T^, *R. ruizarguesonis* UMP1133^T^, *R. sophorae* CCBAU 03386^T^, and *R. trifolii* ATCC 14480^T^ and shared identical *rrs* sequences. The strain PVIM1 grouped with *R. yanglingense* SH22623^T^, *R. loessense* CCBAU 7190B^T^, *R. gallicum* R602sp^T^, *R. mongolense* USDA 1844^T^ with 99.92%, 99.85%, 99.77%, and 97.92% identity, respectively.

Despite that the 16S rRNA gene is widely used as a molecular marker in the taxonomy of prokaryotes, it is not sufficient to differentiate closely related species within the genus *Rhizobium* since different type strains share identical *rrs* sequences^6,60^. In agreement with previous studies, our results showed that *R. anhuiense*, *R. laguerreae*, *R. hidalgonense* FH14^T^, *R. ruizarguesonis* UMP1133^T^ and *R. sophorae,* as well as *R. ecuadorense* and *R. pisi* shared identical *rrs* sequences^8,13,61,62^.

### Multilocus sequence analysis of housekeeping genes

To clarify the 16S rRNA results, multilocus sequence analysis (MLSA) was performed using the housekeeping genes *recA*, *atpD*, *gyrB*, and *glnII* that have widely been used for delineation of *Rhizobium* species as well as for the identification of common bean nodulating rhizobia^14,15,46,47,63,65^. Ribeiro et al. (2009) described a useful MLST scheme for the identification and classification of rhizobial microsymbionts of common bean (*Phaseolus vulgaris* L.) by using housekeeping and symbiotic genes. Tong et al. (2018) demonstrated that a 97.36% threshold in MLSA of three housekeeping genes (~ 1055 bp), was concordant with the 95% ANI threshold for rhizobial species definition. Interestingly, recent genomic and phylogenomic studies have shown that several *Rhizobium* species are organized in well-defined genome clusters with ANI values > 96%, whereas others displayed a continuum of diversity with ANI values > 88%^67,68^. These findings indicated that a default ANI cut-off cannot be applied across all *Rhizobium* species and even more a general threshold for rhizobial species delineation in MLSA cannot be specified as we also pointed out previously^69^. Although phylogeny based on three core genes is not as accurate as the entire genome, ML analysis of few genes can still offer a demonstration for the taxonomic status of rhizobial strains.

In the present study, partial fragments of *recA*, *atpD*, *gyrB,* and *glnII* were amplified from all representative isolates. The number of parsimony-informative sites for every selected gene was estimated within the test *Rhizobium* taxa to find those who were the most phylogenetically informative. In our analysis, *gyrB* had the best percentage of parsimony-informative characters (29.12%), as previously reported^64^, followed by *recA* (25.54%), *atpD* (23.13%), and *glnII* (21.5%).

Gene sequences for *Rhizobium* type/reference strains were retrieved from the GenBank and correctly trimmed. The lengths of the alignments used were 462 bp, 441 bp 594 bp, and 465 bp for *recA*, *atpD*, *gyrB*, and *glnII*, respectively. Phylogenetic trees based on four individual housekeeping genes were constructed and the percentage identity of each gene was also calculated (Supplementary Figs. S3–S6, Supplementary Tables S3–S4). The lengths of the alignments used were 462 bp, 441 bp 594 bp, and 465 bp for *recA*, *atpD*, *gyrB*, and *glnII*, respectively.

**Figure 2:**
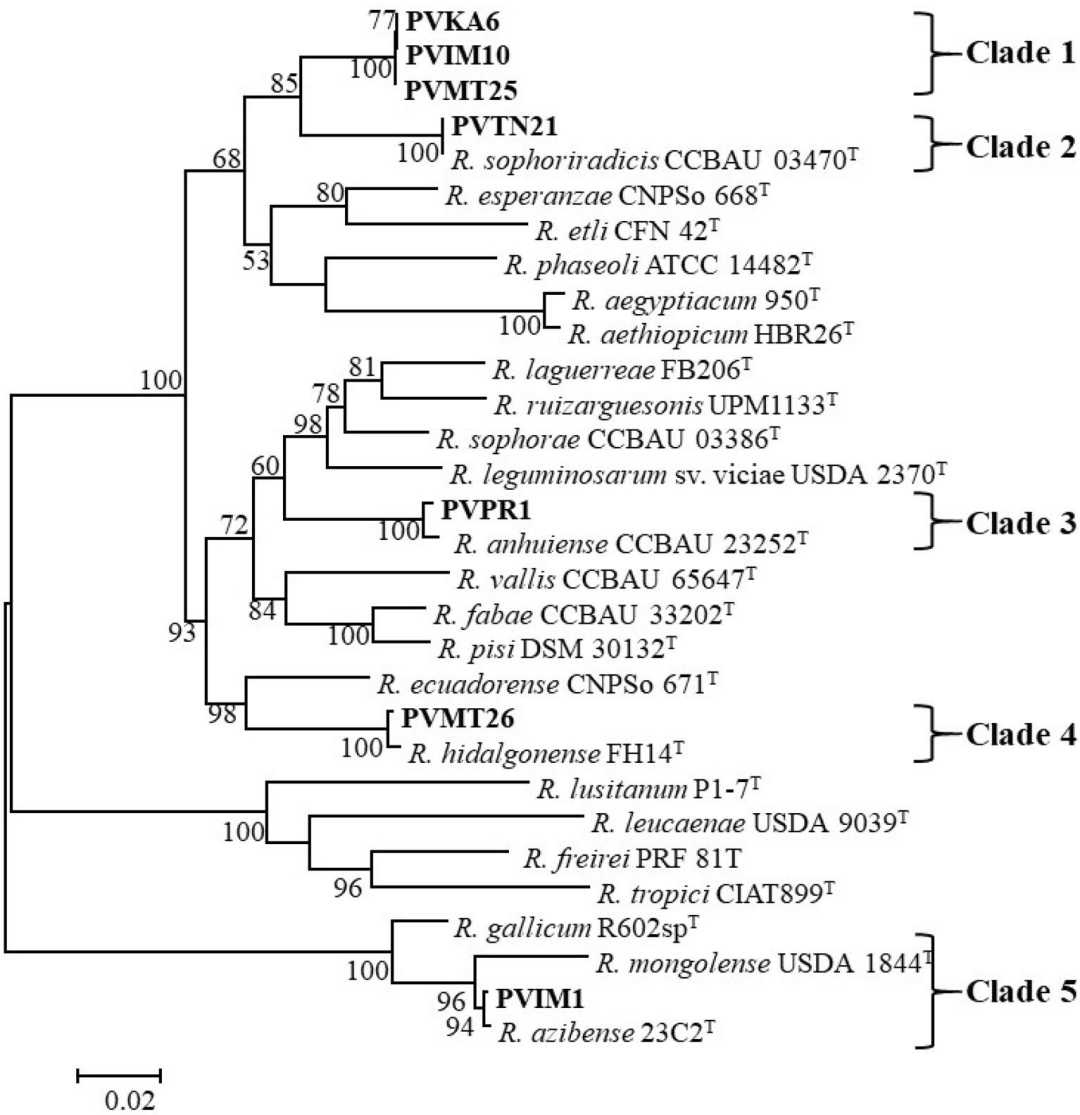
Maximum likelihood phylogenetic tree based on partial concatenated sequences of *recA*, *atpD*, *gyrB,* and *glnII* (with a total of 1962 positions) showing taxonomic relationships of the studied strains and representative related type species. Strains isolated in the present study are shown in boldface and type strains are indicated by superscript “T”. GenBank accession numbers of the sequences are given in Supplementary Figs. S3–S6 and Supplementary Table S2. Bootstrap values (greater than 50%) were calculated for 500 replications and are shown at the nodes. The scale bar shows the number of nucleotide substitutions per site. Phylogenetic analysis was conducted in MEGA 6^104^ (https://www.megasoftware.net/) using the maximum likelihood algorithm with the General Time Reversible model plus Gamma rate distribution plus invariant site (GTR + G + I). The genus names are abbreviated as follows: *R.*, *Rhizobium*.

The analysis of the concatenated sequences of housekeeping genes *recA*, *atpD*, *gyrB*, and *glnII* provided more robust phylogenies of the test strains and congruent with those of the individual gene trees (Fig. 2, Supplementary Figs. S3–S6). The test strains were grouped into five well-supported clades containing defined *Rhizobium* species, except for clade 1 that included the strains PVMΤ25, PVKA6, and PVΙΜ10 originated from three different geographical regions of Greece (Fig. 2, Table 1). Phylogenetic analysis showed that the above strains belong to a wider cluster containing species nodulating *P. vulgaris*, while the closest relative was *R. sophoriradicis* CCBAU 03470^T^ sharing 95.2% identity (Supplementary Table S5). This identity value was lower than those found among *Rhizobium* type strains analysed in the dataset of the present study (Supplementary Table S5). In our pairwise analysis, four pairs of *Rhizobium* type strains showed identity values in the *recA*-*atpD*-*glnII*-*gyrB* concatenated sequences higher than 95.2%, which were presented between the pairs of *R. azibense* 23C2^T^ and *R. mongolense* USDA 1844^T^ (97.25%), *R. gallicum* R602sp^T^ and *R. azibense* 23C2^T^ (96.69%), *R. pisi* DSM 30132^T^ and *R. fabae* CCBAU 33202^T^ (97.6%), *R. aethiopicum* HBR26^T^ and *R. aegyptiacum* 950^T^ (99.24%). These results, together with the position of PVMΤ25, PVKA6, and PVΙΜ10 in the phylogenetic tree suggested that they might constitute a putative novel genospecies within *Rhizobium*. Previously, MLSA and whole-genome analyses defined 25 species or genospecies among the bean-nodulating rhizobia, while species affiliations for some previously named strains were reassigned^6^. Comparison of our strains with the defined genospecies and those isolated previously from bean root-nodules in various countries was also performed to determine their relationships. Since not all gene sequences were available for all strains, a concatenated phylogenetic tree based on the *recA* and *atpD* sequences was constructed (Supplementary Fig. S7). To avoid confusing the reader, in our analysis the grouping of strains taken from the literature did not correspond to the given species names at the time of their deposition, since many bean-nodulating strains were inaccurately assigned at the species level and therefore misnamed due to weak characterization. Interestingly, our isolates were closely related (> 99.9%) to those of *Rhizobium* sp. M1 and M10 isolated from nodules of *P. vulgaris* in China^70^. Recently, the latter two strains were assigned to an unidentified genospecies named as *Rhizobium* sp. I, based on genomic data^6^. Moreover, strains possibly belonging to the genospecies *Rhizobium* sp. I have also been isolated from nodules of *P. vulgaris*, including *Rhizobium* sp. 1648, 1652, and 1706 from China^65^, CTG-412 and CTG-419 from Turkey^71^, L1, B1 and G2 from Iran^5^, GR12 from Spain^44,72^*, Rhizobium* sp. 9 T and 13 T from Croatia^73^. The strains of clade 1 are closely related to each other with identity values above 98.76% and along with our isolates may belong to a new species within *Rhizobium*.

The isolate PVTN21, representing 23 strains isolated from Tinos island of the Aegean Sea and Metsovo, displayed 100% *recA*-*atpD*-*gyrB*-*glnII* nucleotide identity to *R. sophoriradicis* CCBAU 03470^T^, isolated from the root nodule of the medicinal legume *Sophora flavescens* in China^20^ and thus was unambiguously identified as *R. sophoriradicis* (Table 1). According to the *recA*-*atpD* phylogeny (Supplementary Fig. S7), PVTN21 was phylogenetically related to several strains isolated from *P. vulgaris* nodules, such as the strains JJW1, L101, 1587, 1617 and 1532 from China^6,65,70^, NAK368 and NAK378 from Kenya^74^, RHM67 and RHM19 from Morocco^63^, Kim5 from USA^75^, IE4803, IE950, IE4874, and IE4794 from Mexico^43^ and CTG-423 and CTG-430 from Turkey^71^. All strains were grouped in a well-supported cluster (Clade 2) containing *R. sophoriradicis* CCBAU 03470^T^ as well as the strains JJW1, L101, Kim5, and IE4803, which were recently assigned to *R. sophoriradicis* based on genomic data^6^. Therefore, all strains of clade 2 should be assigned to *R. sophoriradicis*. To the best of our knowledge, it is the first time that strains belonging to *R. sophoriradicis* were found in European soils. The wide distribution of *R. sophoriradicis* in *P. vulgaris* nodules all over the world suggests that this species is likely well adapted to different environmental conditions and various bean varieties.

The strain PVPR1, representing five isolates from one region (Table 1), was grouped in clade 3 along with *R. anhuiense* CCBAU 23252^T^ and displayed 99.4% *recA*-*atpD*-*gyrB-glnII* sequence identity (Fig. 2, Table 1, Supplementary Table S5). *R. anhuiense* CCBAU 23252^T^ has been originally isolated from nodules of *Vicia faba* in China and formed ineffective nodules with *P. vulgaris*^76^. However, strains closely related to *R. anhuiense* have been previously isolated from bean nodules and clustered in the same clade (Supplementary Fig. S7), including the strains Y27, S10, J3, JX3 from China^70^ recently assigned to *R. anhuiense*^6^, *Rhizobium* sp. 1627, L6, L13, NC10, M8 also from China^65,70^, CTG-416 from Turkey^5,71^ and LPA1410 from Spain^47^. The strains of clade 3 shared *recA*-*atpD* identity above 99% supporting their affiliation to *R. anhuiense*.

The strain PVMT26, representing seven isolates from one region (Table 1), showed high sequence relatedness to *R. hidalgonense* FH14^T^ in all individual gene phylogenies, with identity values ranging from 99.3 to 100%, and in combined sequences of the four genes (99.6%) (Fig. 2, Supplementary Figs. S3–S6, Supplementary Tables S3–S4). Although this type strain was isolated from nodules of *Phaseolus vulgaris* grown in Mexico^77^, it did not form nodules on its original host *P. vulgaris* and other tested legumes evidenced the loss of its nodulation ability^13^. Despite that *nodC* gene was not amplified from the strain FH14^T^, it is present in the genome sequence of FH14^T^ (NZ_LODW01000075). Strains closely related to *R. hidalgonense* have also been isolated from nodules of *Phaseolus vulgaris* grown in Spain (LBM1212, LBM1123, LCS0303, LCS0401, LCS0411, LEV0613 and RPVR24)^46,47^, Mexico (NH05)^77^, China (CCBAU 65761)^65^, Iran (Hm1)^5^, Kenya (NAK 327, 321, 334)^74^, and Croatia (25 T and 26 T)^73^. Noteworthy, strains closely related to *R. hidalgonense* have also been isolated from other legumes including *Acacia gummifera*^78^, *Indigofera arrecta* in Ethiopia^3^, *Trifolium* spp. in Ethiopia^79^, *T. semipilosum* in Kenya^80^, *Vicia faba* in Ethiopia and China^78,81^. The concatenated analysis of *recA*-*atpD* showed that all these strains formed a highly bootstrapped cluster with *R. hidalgonense* FH14^T^ and displayed high nucleotide identities of *recA*-*atpD* (> 99.4%). Therefore, several strains previously named as *R. leguminosarum*, such as LBM1212, LBM1123, LEV0613, WSM2012, NH05, and CCBAU 65761, or *Rhizobium* sp., such as NAK 327, 321, 334, LCS0401, LCS0411, and RPVR24 might be reclassified in the future as *R. hidalgonense* taking into account phenotypic and chemotaxonomic data.

Phylogenetic analysis based either on the individual or concatenated gene trees showed that PVIM1, representing seven isolates (Table 1), was clustered together with *Rhizobium azibense* 23C2^T^, isolated from common bean nodules in Tunisia^7,36^. Based on the pair-wise comparisons of concatenated sequences of four genes, PVIM1 displayed 99.75% identity to *R. azibense* 23C2^T^ and consequently was assigned to this species (Fig. 2, Supplementary Table S5). Strains belonging to *R. azibense* have also been isolated from nodules of *P. vulgaris* (Supplementary Fig. S7), such as IE4868 from Mexico^43^, 8C-3, and GR42 from Spain^7,36,45^. The strain 8C-3 was originally classified as *R. gallicum*^45^ but it was recently reassigned to *R. azibense* based on genomic data^6^. Interestingly, the strains IE4868, 8C-3 and GR42 formed a separate well-supported sub-clade closely related to *R. azibense* 23C2^T^ with identity values of *recA*-*atpD* concatenated sequences ranged from 96.1% to 96.4%, while the isolate PVIM1 displayed 99.88% identity. Therefore, the Spanish isolates appeared to be more similar to the Mexican ones, while the Greek isolates were phylogenetically closer to the Tunisian strain suggesting that the two sub-clades may represent distinct lineages within *R. azibense* species with a different origin.

Concerning the distribution of our isolates in different regions of Greece, Clade 1 isolates, possibly belonging to genospecies *Rhizobium* sp. I, were found in three regions with different soil textures (SCL, CL and SL) and pH ranging from 6.9 to 7.9 (Supplementary Fig. S1 and Supplementary Table S6). Interestingly, isolates of Clade 2 belonging to *R. sophoriradicis* were predominant in Tinos (soil SCL, pH 8.1), although one isolate was isolated from another region (Metsovo) with different soil textures (SL) and pH 6.9. Despite that Clade 3, 4, and 5 isolates were identified solely in Preveza, Metsovo, and Imathia, respectively, these findings could not rule out the existence of similar isolates in other regions if more isolates were examined or genomic approaches were used. Therefore, the present study cannot provide conclusive evidence for the association of the rhizobial diversity with the edaphic parameters or host genotypes at our sampling sites. To define the factors influencing the distribution of different species or genospecies in Greek soil, further studies are required.

### Phylogenetic analysis of symbiosis genes *nodC* and *nifH*

Currently, the *nodC* gene is commonly used to define symbiovars within rhizobial species. *P. vulgaris* is considered to be a promiscuous host since it can be nodulated by different rhizobial species and symbiovars^1,2^. At least thirty rhizobial species and eight symbiovars have been reported to nodulate common bean so far^2,31,32^. However, most bean-nodulating rhizobia, regardless of their species affiliation, belong to sv. phaseoli, which also exclusively nodulates *P. vulgaris*^12,82^. Previously, the sv. phaseoli was divided into three sub-clades, representing different alleles of *nodC* designated α, γ-a, and γ-b^5,39,74^. The γ *nodC* allele is considered to be the most widely distributed worldwide, implying a distribution of this allele together with bean seeds from their American distribution centers^39,46,82,83^.

**Figure 3:**
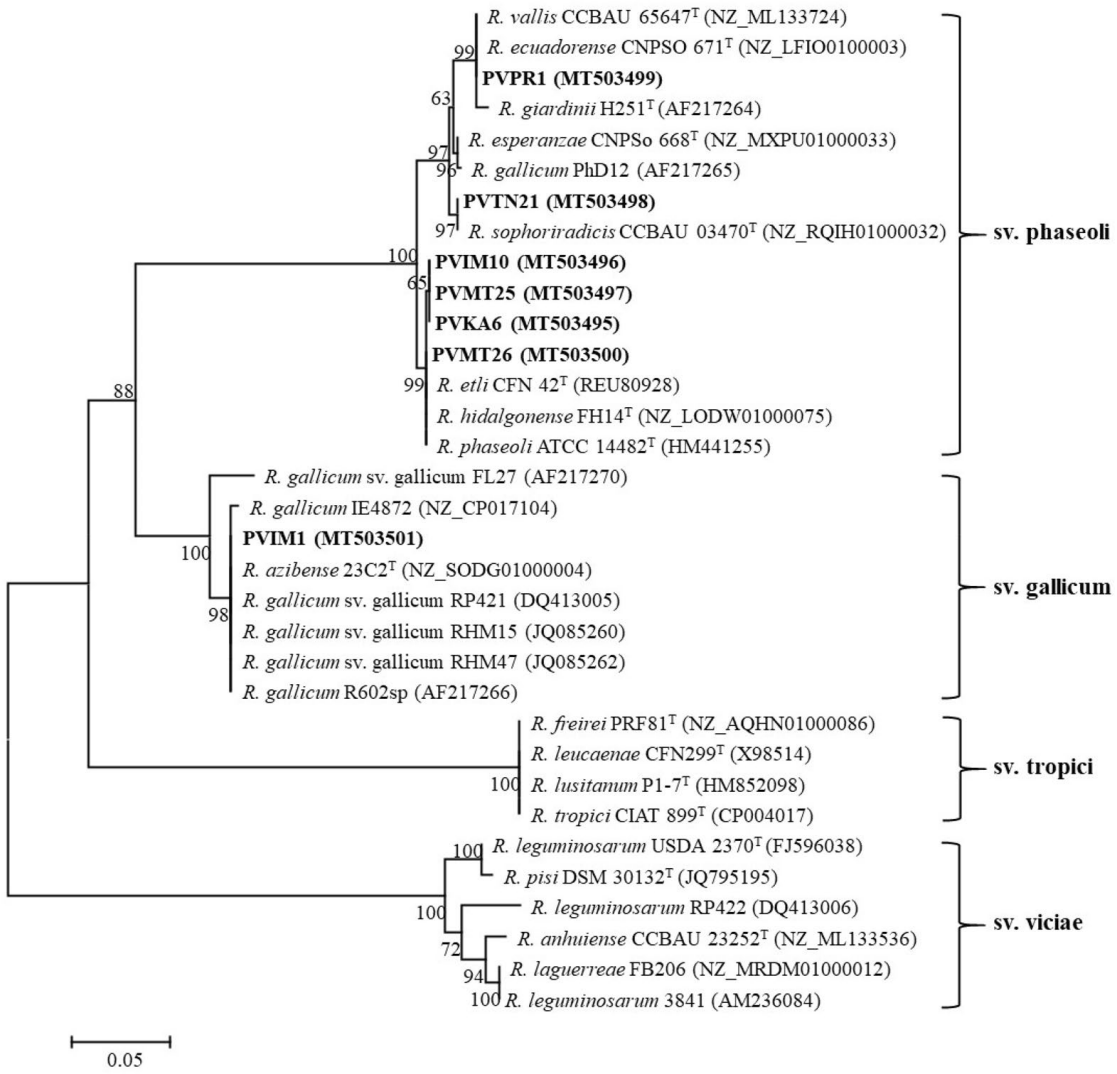
Maximum likelihood phylogenetic trees based on 543-bp alignment of the *nodC* nucleotide sequences showing the symbiovars to which the strains isolated in this study belong. The taxonomic relationships of the studied strains and the closest type strains of *Rhizobium* species are shown. Strains isolated in the present study are shown in boldface and their accession numbers are given in Supplementary Table S2. Type strains are indicated by superscript “T” and GenBank accession numbers of their sequences are indicated within parentheses. Bootstrap values (greater than 50%) were calculated for 500 replications and are shown at the nodes. The scale bar shows the number of nucleotide substitutions per site. Phylogenetic analysis was conducted in MEGA 6^104^ (https://www.megasoftware.net/) using the maximum likelihood algorithm with the Tamura 3-parameter model plus invariant site (T92 + I). The genus names are abbreviated as follows: *R.*, *Rhizobium.*

**Figure 4:**
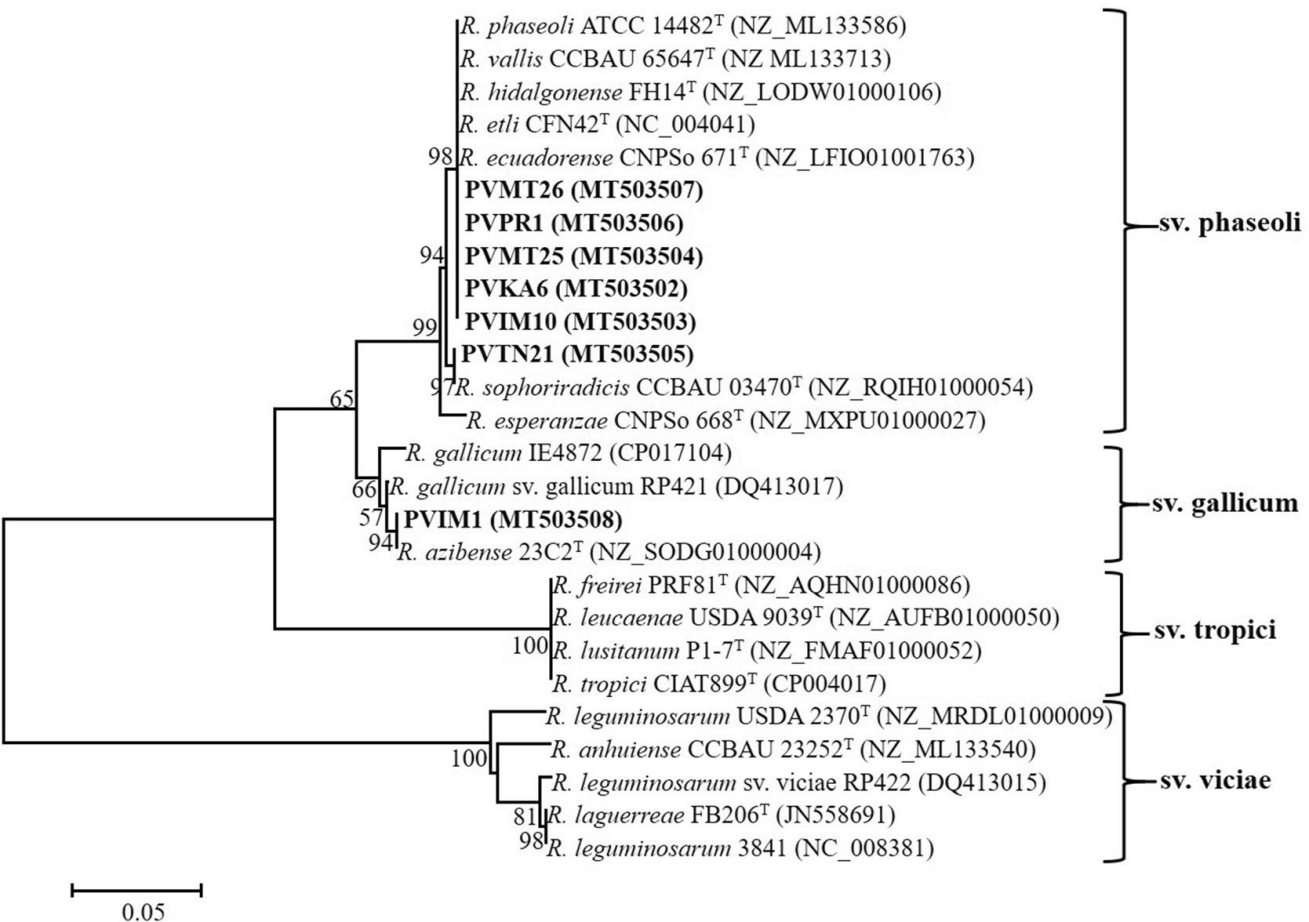
Maximum likelihood phylogenetic trees based on 726-bp alignment of *nifH* nucleotide sequences. The taxonomic relationships of the studied strains and the closest type strains of *Rhizobium* species are shown. Strains isolated in the present study are shown in boldface and their accession numbers are given in Supplementary Table S2. Type strains are indicated by superscript “T” and GenBank accession numbers of their sequences are indicated within parentheses. Bootstrap values (greater than 50%) were calculated for 500 replications and are shown at the nodes. The scale bar shows the number of nucleotide substitutions per site. Phylogenetic analysis was conducted in MEGA 6^104^ (https://www.megasoftware.net/) using the maximum likelihood algorithm with the Tamura 3-parameter model plus Gamma rate distribution (T92 + G). The genus names are abbreviated as follows: *R.*, *Rhizobium.*

**Figure 5:**
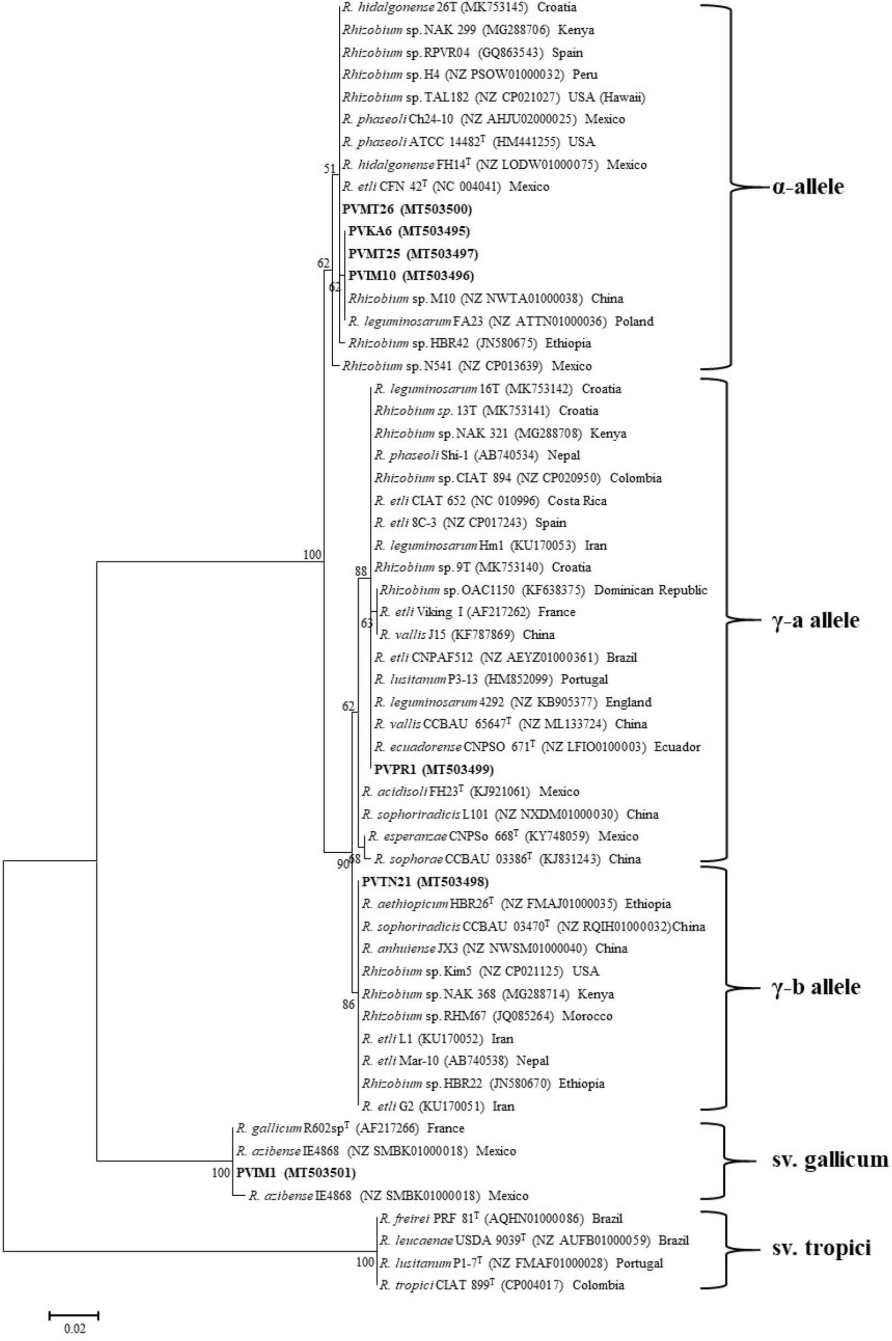
Maximum likelihood phylogenetic tree based on *nodC* gene sequences (405 bp) showing phylogenetic relationships between the strains of the symbiovars phaseoli and gallicum isolated in this work and those in other geographical locations. Strains isolated in the present study are shown in boldface and type strains are indicated by superscript “T”. GenBank accession numbers of the sequences are indicated within parentheses. Bootstrap values (greater than 50%) were calculated for 1000 replications and are shown at the nodes. The scale bar shows the number of nucleotide substitutions per site. Phylogenetic analysis was conducted in MEGA 6^104^ (https://www.megasoftware.net/) using the maximum likelihood algorithm with the Tamura 3-parameter model (T92). R., *Rhizobium*.

Partial nucleotide sequences of *nodC* and *nifH* were amplified and sequenced for all representative strains and their phylogenetic trees are shown in Figs. 3 and 4, respectively. In the *nodC* and *nifH* trees, most Greek isolates were placed into two well-supported clades that corresponded to symbiovars phaseoli and gallicum. The inclusion of representative strains carrying different *nodC* alleles from previous works in our phylogenetic analysis allowed us to define the *nodC* alleles of the studied strains (Fig. 5). Interestingly, isolates belonging to sv. phaseoli were clustered into three subgroups coincident with the previously described alleles α, γ-a, and γ-b^74^.

The α allele was found in the Greek strains closely related to *R. hidalgonense* and *Rhizobium* sp. I. The α allele is considered to have originated in America and was distributed to Europe and other continents with bean seeds^39,48,64,83^. The strain PVMT26, assigned as *R. hidalgonense*, carried the α *nodC* allele, which was identical to that of the type strains *R. hidalgonense* (Mexico), *R. etli* (Mexico), and *R. phaseoli* (USA)*,* and displayed 99.8% identity to the putative new lineages PVIM10, PVMT25, and PVKA6 (Fig. 3). The α allele has also been found in strains of the undescribed species *Rhizobium* sp. I (M1, M10, H4, 1648, 1652, NAK 299, 26 T), *Rhizobium* sp. II (N541), *Rhizobium* sp. IX (FA23), *R. esperanzae* (TAL182), *R. phaseoli* (NAK 299, Ch24-10) and *Rhizobium* sp. RPVR04 and HBR42 (Fig. 5). For simplification, not all strains carrying the α allele were included in the *nodC* phylogenetic tree. The identities of α *nodC* alleles found in various strains isolated from various countries ranged between 99.2 and 100%. In European soils, the α allele has been found in strains affiliated to *R. hidalgonense* in Croatia^73^, *R. etli* in Spain^46^, and *R. leguminosarum* in Poland^84^.

The strain PVPR1 assigned to *R. anhuiense* harbored the γ-a *nodC* allele, which was identical to those of the type strains *R. vallis*, and *R. ecuadorense* isolated from bean nodules in China and Ecuador, respectively^8,23^*.* The γ-a allele was also harbored by the type strains of *R. acidisoli* (Mexico), *R. esperanze* (Mexico), and *R. sophorae* (China) sharing 99–99.5% identity with that of PVPR1. The γ-a allele is also present in strains belonging to other species, such as *R. etli*, *R. leguminosarum, R. lusitanum, R. phaseoli, and R. sophoriradicis* with identity values among strains ranging from 97.2 to 100% (Fig. 5). Therefore, this allele was not only found in strains isolated from *P. vulgaris* nodules in various countries from all continents but also was the most prevalent within the rhizobial species nodulating common bean. In European soils, the γ-a *nodC* allele is the most frequent among bean-nodulating rhizobia regardless of the species to which they belong^12,18,39,46–48,73,82,85^. Considering that the sv. phaseoli evolved with common beans in America^39,86^ and probably disseminated worldwide along with bean seeds^2,87^, it is possible that native rhizobia in various countries have acquired symbiotic genes typical of sv. phaseoli through horizontal gene transfer in the rhizosphere or within nodules^88,89^.

The Greek strains identified as *R. sophoriradicis* and represented by PVTN21 harbored the γ-b allele, which is present in the type strains of *R. aethiopicum* and *R. sophoriradicis* (Fig. 5). Noteworthy, all γ-b *nodC* alleles found in various strains were identical (100%) and were found in Asia (China, Iran), Africa (Ethiopia, Kenya, Morocco), and America (USA, Mexico)^5,63,65,70,74,90,91^. Most strains carrying this allele were closely related to *R. sophoriradicis* (Kim5, IE4803, RHM67, RHM19, NAK368, NAK378, NAK387, L1, S1, G1, B1, 1706, 1587, 1617, and 1532), except for strain L101 that carried the γ-a allele and the strain IE4771 harbored a *nodC* gene similar to the sv. gallicum. Moreover, this allele is also present in *R. anhuiense* strains, such as JX3 Y27, S10, C15, J3 from China^6,70^, in *Rhizobium* sp. I (e.g. *Rhizobium* sp. G2) from Iran^5^ and in *Rhizobium* sp. strains Mar-10 and HBR22 from Nepal and Ethiopia, respectively^90,92^. Therefore, this allele seems to be restricted to a few rhizobial species with prevalence in *R. sophoriradicis*. To the best of our knowledge, this is the first time that the γ-b allele was found in European soils and within isolates assigned to *R. sophoriradicis*.

Finally, strains identified as *Rhizobium azibense* and represented by PVIM1 harbored *nodC* genes identical (100%) to sv. gallicum, which is present in *R. azibense* 23C2^T^, and *R. gallicum* R602sp^T^ isolated from bean nodules in Tunisia and France, respectively ^7,12,36^. However, the *R. azibense* strains 8C-3, and GR42, isolated from bean nodules in Spain belong to sv. phaseoli harboring the γ-a allele^7,44,45,93^ as shown in Fig. 5. Strains belonging to sv. gallicum have also been isolated from common bean in Austria^51^, Tunisia^36,94^, Morocco^63,95^, and Mexico^43,51^. Previously, it was suggested that the occurrence of sv. gallicum in European soils may be correlated with the introduction of common beans along with their seed-borne symbionts from America^61^. Interestingly, the European and African strains harbored identical *nodC* gene sequences and to that of the type strain *R. gallicum* R602sp^T^, while the Mexican isolates IE4868, FL27, and IE4771 carry more diversified *nodC* genes with identity values 99.51%, 96.54%, and 93.83%, respectively. Although the Mexican isolate FL27 was previously demonstrated to be a poor N fixer in common bean nodules^96^, it remains to be investigated whether the European and African strains nodulating common bean possess a better symbiotic efficiency since they carry more divergent *nodC* genes.

Noteworthy, the sv. gallicum has also been reported to effectively nodulate legumes belonging to the genera *Leucaena*, *Macroptilium*, *Onobrychis*, *Sesbania*, *Caliandra*, *Gliricidia*, *Leucaena,* and *Piptadenia*^12,26,44,45,52,78,97–99^. The *nodC* gene sequences of our isolates were also identical to those found in sv. gallicum strains isolated from nodules of other legumes, such as the strains *Rhizobium* sp. AC91a from *Calliandra calothyrsus* in Ethiopia^78^, *R. tarimense* AS1-101a and SPT1 from *Ammopiptanthus* in China, and *Rhizobium* sp. UPRM 8060 from *Piptadenia flava* in Puerto Rico^100^. For simplification, not all strains from other legumes were included in the *nodC* phylogenetic tree. The wide distribution of sv. gallicum in different continents in combination with its broad host range and its presence in different rhizobial species makes it a promising multi-host inoculant.

Phylogenetic analysis based on partial *nifH* sequences (726 bp) grouped the isolates into two clades that corresponded to symbiovars phaseoli and gallicum (Fig. 4). The phaseoli clade consisted of two sub-clades with an identity 99.3%. One sub-clade included the isolates PVIM10, PVKA6, PVMT25, PVMT26, and PVPR1, which shared identical *nifH* sequences to those of *R. hidalgonense* FH14^T^, *R. phaseoli* ATCC 14482^T^, *R. etli* CFN42^T^, *R. ecuadorense* CNPSO 671^T^, and *R. vallis* CCBAU 65647^T^. Strain PVTN21 was separately clustered along with *R. sophoriradicis* CCBAU 03470^T^ displaying identical *nifH* sequences. Strain PVIM1 had an identical *nifH* sequence to that of *R. azibense* 23C2^T^ and formed a clade that corresponded to symbiovar gallicum. Overall, the phylogenetic analysis of *nifH* was congruent with that of *nodC* phylogeny.

## Conclusions

In summary, the present study provides the first analysis on the phylogenetic diversity of indigenous rhizobia nodulating *P. vulgaris* in Greece by identifying them at the species and symbiovar level. Strains were affiliated to *R. anhuiense*, *R. azibense*, *R. hidalgonense, R. sophoriradicis*, and to a putative new genospecies consisting of various strains all over the world and provisionally named as *Rhizobium* sp. I^6^. Most strains belonged to symbiovar phaseoli carrying the α-, γ-a and γ-b alleles of *nodC* gene, while few of them belonged to symbiovar gallicum. To the best of our knowledge, it is the first time that strains assigned to *R. sophoriradicis* and harbored the γ-b allele were found in European soils. All strains formed effective symbioses with bean plants, suggesting that they are true symbionts of common bean. The analysis of the symbiovar phaseoli *nodC* alleles is congruent with previous findings in other European countries suggesting the American origin of sv. phaseoli. Moreover, the presence of *nodC* alleles in diverse rhizobial strains regardless of the species to which they belong raises the possibility that local rhizobia have acquired symbiosis genes via lateral gene transfer in the rhizosphere or within nodules. However, the *Rhizobium azibense* isolates were closely related and grouped together with African strains in both MLSA and *nodC* phylogenies suggesting their common evolutionary histories. Consequently, the current study increases the knowledge of the diversity, geographic distribution, and evolution of common bean-nodulating rhizobia in European soils and further provides a natural resource for the selection of highly efficient rhizobia that are more competitive and adapted to the local conditions.

## Μethods

### Nodule and soil sampling

Nodules were collected from local common bean varieties grown in five different geographical regions of Greece, namely as Imathia, Metsovo, Preveza, Tinos, and Karpathos (Supplementary Fig. S1). The sampling sites were located in fields with no history of rhizobial inoculation. The soil samples were slightly acidic to alkaline, with pH range 6.9 to 8.1.

### Isolation and purification of nodules and rhizobial strains

Four nodules per plant were randomly selected from four plants of each region and at least three isolates were retained from each nodule. A great number of isolates were non-nodulating bacterial strains which were probably nodule endophytes or contaminants and they were not analyzed further. Finally, a total of 50 rhizobial strains were isolated in pure culture. Standard routine laboratory techniques were applied for the isolation of strains from the nodules^101^. Briefly, the nodules were surface disinfected by immersion in 70% ethanol for 60 s and then in 3–5% (v/v) solution of sodium hypochlorite for 2–4 min and were washed six times with sterile ddH_2_O. To check the absence of surface contamination, sterilized nodules were rolled over yeast-mannitol agar (YMA) plates^101^ and aliquots of water from the last washing step were also spread on YMA plates and incubated at 28 °C for 2–5 days. Sterilized nodules were crushed in a drop of sterile distilled water and the nodule juice was streaked onto YMA plates and incubated under the same conditions as the control plates. Only nodules without any contaminants were considered for the isolation of rhizobial strains. Single colonies were subsequently purified by repeated streaking on YMA medium supplemented with Congo red until pure cultures of the isolates were obtained. Cultures of pure isolates were maintained in 20% glycerol–YMA broth at − 80 °C.

### Nodulation tests

The nodulation capability of each isolate was tested by inoculating seedlings of its original host grown in a greenhouse. Seeds were surface sterilised in 3% sodium hypochlorite for 10 min and rinsed six times. Surface-sterilized seeds were germinated on moist sterile filter paper in the dark at 22 °C for 3–4 days and then transferred to 250 ml pots containing vermiculite and watered with 0.5Χ Hoagland nutrient solution without nitrogen^102^. Each seedling was inoculated with 1 ml of rhizobial suspension (∼10^9^ cells ml^−1^). Three replicates were performed per isolate and plants were grown in greenhouse. Unfertilized and uninoculated seedlings were included as negative controls and uninoculated, nitrogen fertilized (5 mM KNO_3_) seedlings were used as positive controls. Six weeks after inoculation, one nodule per plant was excised and rhizobia were re-isolated as described above and their identity was confirmed by BOX-PCR fingerprinting. Nodulation capacity was recorded as positive (Nod+) when nodules were present and negative (Nod−) if were absent. Nitrogen fixation was considered effective when nodules were pink (Fix+) and ineffective if nodules were white (Fix−).

### DNA isolation and BOX-PCR fingerprinting

Total template DNA was extracted from each isolate using the PureLink™ Genomic DNA kit (Thermo Fisher Scientific), according to manufacter’s instructions. BOX-PCR fingerprint analysis was performed by using the BOX A1R primer (Supplementary Table S1)^103^. PCR reactions were carried out in a final volume of 25 µl containing 100 ng of genomic template DNA, 1X reaction buffer (75 mM Tris–HCl pH 8.8, 20 mM (NH_4_)_2_SO_4_, 0.01% Tween 20, 2 mM MgCl_2_), 0.2 mM dNTPs, 2.5 U DreamTaq DNA polymerase (Thermo Fisher Scientific), and 50 pmol of primer. The PCR conditions were: initial denaturation at 94 °C for 7 min, 30 cycles of denaturation at 94 °C for 1 min, annealing at 50 °C for 1 min, and extension at 65 °C for 8 min. PCR reactions were terminated by a final extension at 65 °C for 16 min. All PCR products were separated by electrophoresis in 1.5% agarose gels containing 0.5 µg ml^−1^ ethidium bromide at 60 V for 3.0 h. A molecular marker 1 kb DNA Ladder, (Invitrogen) was included on the left. The gels were scanned with the GelDoc system (Bio-Rad, Hercules, CA).

### PCR amplification and sequencing

The DNA fragments of 16S rRNA, *recA* (DNA recombination protein*)*, *atpD* (ATP synthase subunit beta), *gyrB* (DNA gyrase B) and *glnII* (glutamine synthetase II) were amplified by PCR, using the primer pairs described in Supplementary Table S1. PCR amplification and sequencing were carried out as previously described^69^. Primers taken from the literature or designed in the present study were slightly modified in such a way to include at their 5′ ends either T7 or SP6 primer sequence to facilitate direct sequencing of the amplicons. Each PCR mixture contained the following: approximately 50 ng genomic DNA, 20 pmol each primer, 200 µM dNTPs (Invitrogen), Phusion High Fidelity DNA polymerase (Thermo Fisher Scientific), and the respective 10X polymerase buffer in a final reaction volume of 50 µl. The PCR conditions for the amplification of each gene fragment are described in Supplementary Table S1. PCR products from the aforementioned genes were purified using the PureLink™ Quick Gel Extraction kit (Thermo Fisher Scientific). Purified DNA fragments were directly sequenced on both strands using the standard primers attached in the corresponding primer sequences. All PCR products were commercially sequenced by CEMIA (cemia.eu), Greece.

### Phylogenetic analyses

The sequences of *rrs* genes were compared with those of bacterial type strains using the EzTaxon-e server (http://eztaxon-e.ezbiocloud.net). BLAST searches were done at the National Center for Biotechnology Information (NCBI) server using BLASTN (http://www.ncbi.nlm.nih.gov/blast). Sequences from closely related type strains, as listed on the List of Prokaryotic Names with Standing in Nomenclature (LPSN) (www.bacterio.net), and reference strains were retrieved for phylogenetic analyses from the GenBank database (http://www.ebi.ac.uk/Tools/sss/fasta/nucleotide.html). For pairwise distance matrixes, the multiple sequence alignments were performed using the algorithm CLUSTAL Omega (https://www.ebi.ac.uk/Tools/msa/clustalo/) provided by the European Bioinformatics Institute (EMBL-EBI). For phylogenetic analyses, the partial gene sequences obtained in this study, together with sequences retrieved from GenBank were aligned using the CLUSTALW software in the MEGA 6.0 software package^104^. Phylogenetic trees were constructed using either the neighbor-joining (NJ) or Maximum likelihood (ML) methods in MEGA 6.0 software package. The gene sequences were appropriately trimmed and were concatenated. The best-fit models of nucleotide substitution were determined in MEGA 6 and the most appropriate were selected for the construction of ML trees as referred in the figure legends.

### Nucleotide sequence accession numbers

All sequences from common bean isolates were deposited in the GenBank database and the accession numbers are listed in Supplementary Table S2.

### Ethics approval

This article does not contain any studies with human participants and/or animals performed by any of the authors. The formal consent is not required in this study.

## Supplementary Information

Supplementary Information.

## Data Availability

Sequence data that support the findings of this study have been deposited in GenBank (https://www.ncbi.nlm.nih.gov/genbank/) with the accession codes: MT476928-MT476934 and MT503467-MT503508. Sequence data MT503467-MT503508 will be publicly available upon article publication but are available from the corresponding author on reasonable request.
