# Supplementary material for: Genetic characterization at the species and symbiovar level of indigenous rhizobial isolates nodulating Phaseolus vulgaris in Greece
Source: Sci Rep. 2021 Apr 21;11:8674. doi: 10.1038/s41598-021-88051-8 (PMC8060271; doi:10.1038/s41598-021-88051-8)
Supplement: Supplementary file 1 — Supplementary Information. [file 41598_2021_88051_MOESM1_ESM.docx]

**SUPPLEMENTARY MATERIAL**

**Genetic characterization at the species and symbiovar level of indigenous rhizobial isolates nodulating *Phaseolus vulgaris* in Greece**

**Evdoxia Efstathiadou^1^, Georgia Ntatsi^2^, Dimitrios Savvas^2^, Anastasia P. Tampakaki^1*^**

^1^Laboratory of General and Agricultural Microbiology, Department of Crop Science, Agricultural University of Athens, Iera Odos 75, Votanikos, 11855 Athens, Greece.

^2^Laboratory of Vegetable Production, Department of Crop Science, Agricultural University of Athens, Iera Odos 75, Votanikos, 11855 Athens, Greece.

*^*^Correspondence to:*

Anastasia P. Tampakaki, E-mail: [tampakaki@aua.gr](mailto:tampakaki@aua.gr)

ORCID identifier: 0000-0003-4439-0920

**Supplementary Table S1.** Primers and PCR conditions used in this study.

| **Oligonucleotide name** | **Oligonucleotide sequence^1^**  **(5’ to 3’)** | **Gene/Sequence** | **Amplicon size in bp** | **PCR conditions** | **Reference** |
| --- | --- | --- | --- | --- | --- |
| BOX A1R | CTACGGCAAGGCGACGCTGACG | BOX-sequences | Variable sizes | 5 min 95 ◦C, 30 × (30 s 94 ◦C, 1 min 52 ◦C, 8 min 65 ◦C), 16 min 65 ◦C | Versalovic et al., 1994 |
| 16S-F1-T7 | TAATACGACTCACTATAGGGAGAGTTTGATCCTGGCTCAG | 16S rRNA | ~1500 | 5 min 95 ◦C, 30 × (1 min 95 ◦C, 1 min 55 ◦C, 2.0 min 72 ◦C), 10 min 72 ◦C | Modified from Weisburg et al. 1991 |
| 16S-R1-SP6 | GATTTAGGTGACACTATAGAAGGAGGTGATCCAGCC | 16S rRNA |  |  |  |
| *gyrB*340F-T7 | TAATACGACTCACTATAGGGTTCGACCARAAYTCYTACAAGG | *gyrB* | ~700 | 5 min 95 ◦C, 30 × (1 min 95 ◦C, 1 min 58 ◦C, 1.0 min 72 ◦C), 10 min 72 ◦C | Tampakaki et al 2017a |
| *gyrB*1057rhi-R-SP6 | GATTTAGGTGACACTATAGCSAGCTTRTCCTTGGTCTGCG | *gyrB* |  |  | Efstathiadou et al, 2020 |
| *glnII*12F-rhi-T7 | TAATACGACTCACTATAGGGYAAGCTCGAGTAYATYTGGCT | *glnII* | ~650 | 5 min 95 ◦C, 30 × (1 min 95 ◦C, 1 min 56 ◦C, 1.0 min 72 ◦C), 10 min 72 ◦C | Modified from Vinuesa et al 2005 |
| *glnII*689R-rhi-SP6 | GATTTAGGTGACACTATAGTGCATGCCCGRCCRTTCCA | *glnII* |  |  | Modified from Vinuesa et al 2005 |
| *recA*41F-T7 | TAATACGACTCACTATAGGGTTCGGCAAGGGMTCGRTSATG | *recA* | ~700 | 5 min 95 ◦C, 30 × (1 min 95 ◦C, 1 min 56 ◦C, 1.0 min 72 ◦C), 10 min 72 ◦C | Tampakaki et al, 2017a |
| *recA*827R-SP6 | GATTTAGGTGACACTATAGCCATACATGATRTCGAAYTC | *recA* |  |  | Tampakaki et al, 2017a |
| *atpD*-F52-T7 | TAATACGACTCACTATAGGGGTTATCGGCGCCGTYGTSGACGT | *atpD* | ~850 | 5 min 95 ◦C, 30 × (1 min 95 ◦C, 1 min 54 ◦C, 1.0 min 72 ◦C), 10 min 72 ◦C | Efstathiadou et al, 2020 |
| *atpD*-F902-SP6 | GATTTAGGTGACACTATAGGTSGTSGTRATGCGYTCCTGCATC-3’ |  |  |  | Efstathiadou et al, 2020 |
| *nifH*-FP-T7 | TAATACGACTCACTATAGGGTACGGCAAAGGGGGSATCGGCAA | *nifH* | ~850 | 5 min 95 ◦C, 30 × (1 min 95 ◦C, 1 min 55 ◦C, 1.0 min 72 ◦C), 10 min 72 ◦C | Efstathiadou et al, 2020 |
| *nifH*-RPV-SP6 | GATTTAGGTGACACTATAGAGCATGTCYTCVAGYTCYTCCA | *nifH* |  |  | Efstathiadou et al, 2020 |
| *nodC*-F352P-T7 | TAATACGACTCACTATAGGGGTYGGYAAACGGAAGGCACAGAT | *nodC* | ~800 | 5 min 95 ◦C, 30 × (1 min 95 ◦C, 1 min 55 ◦C, 1.0 min 72 ◦C), 10 min 72 ◦C | Efstathiadou et al, 2020 |
| *nodC*mrs-R-SP6 | GATTTAGGTGACACTATAGCGHGACAGCCARTCGCTRTTG | *nodC* |  |  | Tampakaki et al 2017b |

^1^Mixtures of bases used at certain positions are given as: R, A or G; Y, C or T; M, A or C; K, G or T; S, G or C; B,C or G or T; V, A or C or G. Underlined sequences indicate nucleotide sequences of T7 or SP6 sequencing primers.

Efstathiadou, E., Savvas, D. & Tampakaki, A. P. Genetic diversity and phylogeny of indigenous rhizobia nodulating faba bean (*Vicia faba* L.) in Greece. *Syst. Appl. Microbiol.* **43**,126149, DOI: [https://doi.org/10.1016/j.syapm.2020.126149](https://www.sciencedirect.com/science/article/pii/S0723202020301041) (2020).

Tampakaki, A. P., Fotiadis, C. T., Ntatsi, G. & Savvas, D. Phylogenetic multilocus sequence analysis of indigenous slow-growing rhizobia nodulating cowpea (*Vigna unguiculata* L.) in Greece. *Syst. Appl. Microbiol.* **40**,179-189, DOI: [https://doi.org/10.1016/j.syapm.2017.01.001](https://www.sciencedirect.com/science/article/pii/S0723202017300024?casa_token=9acOivor6T8AAAAA:JwV4zQ4C64SIvZVnYFLHDdzceZIwpUwYkc1jhZLzUd567-rcU1heYNOQMVbM4KbrgKzu8K4W) (2017a).

Tampakaki, A. P., Fotiadis, C.T., Ntatsi, G. & Savvas, D. [A novel symbiovar (aegeanense) of the genus](https://www.ncbi.nlm.nih.gov/pubmed/28220509) *[Ensifer](https://www.ncbi.nlm.nih.gov/pubmed/28220509)* [nodulates](https://www.ncbi.nlm.nih.gov/pubmed/28220509) *[Vigna unguiculata.](https://www.ncbi.nlm.nih.gov/pubmed/28220509)* J. *Sci. Food Agric*. **97**, 4314-4325, DOI: [https://doi.org/10.1002/jsfa.8281](https://onlinelibrary.wiley.com/doi/full/10.1002/jsfa.8281?casa_token=32cOVjmZDtcAAAAA%3ABeueu5XAIhBi7Mp_vQbSYw7dmu0ZgOupNZryczLyggLpyj_DlPHAHmV6k3CaaWJvy20vUvbkr9ohlw) (2017b).

Versalovic, J., Schneider, M., de Brulin, F. J.& Lupski, J. R. Genomic fingerprinting of bacteria using repetitive sequence-based polymerase chain reaction. *Methods Mol. Cell Biol.* **5**, 25-40 (1994).

Vinuesa, P., Silva, C., Lorite, M. J., Izaguirre-Mayoral, M. L., Bedmar, E. J. & Martínez-Romero, E. Molecular systematics of rhizobia based on maximum likelihood and Bayesian phylogenies inferred from *rrs, atpD, recA* and *nifH* sequences, and their use in the classification of *Sesbania* microsymbionts from Venezuelan wetlands*. Syst. Appl. Microbiol.* **28**, 702-16, DOI: [https://doi.org/10.1016/j.syapm.2005.05.007](https://www.sciencedirect.com/science/article/pii/S0723202005000883) (2005).

Weisburg, W.G., Barns, S.M., Pelletier, D.A. & Lane, D.J. 16S ribosomal DNA amplification for phylogenetic study. *J. Bacteriol.* **173**, 697–703, DOI: <https://doi.org/10.1128/jb.173.2.697-703> (1991).

**Supplementary Table S2.** GenBank accession numbers of the sequenced genes in this study.

| **Strain** | ***rrs*** | ***recA*** | ***atpD*** | ***gyrB*** | ***glnII*** | ***nodC*** | ***nifH*** |
| --- | --- | --- | --- | --- | --- | --- | --- |
| *Rhizobium* sp. PVKA6 | MT476928 | MT503467 | MT503474 | MT503481 | MT503488 | MT503495 | MT503502 |
| *Rhizobium* sp. PVIM10 | MT476929 | MT503468 | MT503475 | MT503482 | MT503489 | MT503496 | MT503503 |
| *Rhizobium* sp. PVMT25 | MT476930 | MT503469 | MT503476 | MT503483 | MT503490 | MT503497 | MT503504 |
| *R. sophoriradicis* PVTN21 | MT476931 | MT503470 | MT503477 | MT503484 | MT503491 | MT503498 | MT503505 |
| *R. anhuiense* PVPR1 | MT476932 | MT503471 | MT503478 | MT503485 | MT503492 | MT503499 | MT503506 |
| *R. hidalgonense* PVMT26 | MT476933 | MT503472 | MT503479 | MT503486 | MT503493 | MT503500 | MT503507 |
| *R. azibense* PVIM1 | MT476934 | MT503473 | MT503480 | MT503487 | MT503494 | MT503501 | MT503508 |

**Supplementary Table S3.** Identities of *recA* (462 bp) and *atpD* (441 bp) nucleotide sequences among *Rhizobium* strains. The closest type strains are shown in bold.

**Supplementary Table S4.** Identities of *gyrB* (594 bp) and *glnII* (465 bp) nucleotide sequences among *Rhizobium* strains. The closest type strains are shown in bold.

**Supplementary Table S5.** Identities of concatenated sequences of *recA, atpD, gyrB,* and *glnII* among *Rhizobium sp.* strains. The closest type strains are shown in bold.

**Supplementary Table S6.** Distribution and abundance of isolates clustered in different clades in sampling sites.

|  | **Clade 1** |  | **Clade 2** | **Clade 3** | **Clade 4** | **Clade 5** |
| --- | --- | --- | --- | --- | --- | --- |
| **Imathia** | 5 |  |  |  |  | 7 |
| **Karpathos** | 2 |  |  |  |  |  |
| **Metsovo** | 1 |  | 1 |  | 7 |  |
| **Preveza** |  |  |  | 5 |  |  |
| **Tinos** |  |  | 22 |  |  |  |
| **Total isolates** | 8 |  | 23 | 5 | 7 | 7 |

**
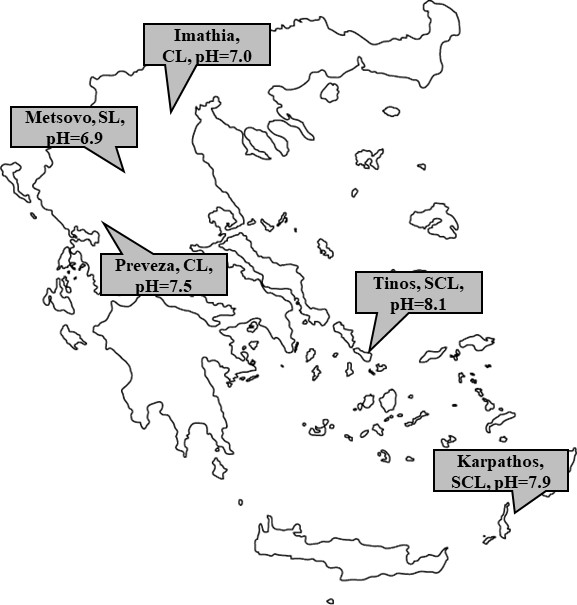
**

**Supplementary Figure S1.** Simplified map of Greece showing the sampling sites of common bean-nodulating rhizobia. The coordinates (Latitude/Longitude) were as follows: 1: Imathia, N40°29'19.4“/E22°11'13.0"; 2: Metsovo, Ν39°43'40.0"/Ε21°03'40.4"; 3: Preveza, Ν39°16'07.8"/Ε20°51'18.7"; 4: Tinos island, Ν37°34'26.8"/Ε25°10'05.7"; 5: Karpathos island, Ν35°44'14.3"/Ε27°10'58.2". The soil texture class and pH of the sampling sites are indicated within grey boxes. CL: clay-loam; SL: sandy-loam; SCL: sandy-clay-loam. Greece map was created by Vemaps.com (https://vemaps.com/) and additions were made for the purpose of this work with PowerPoint-Office 365 by A. Tampakaki.


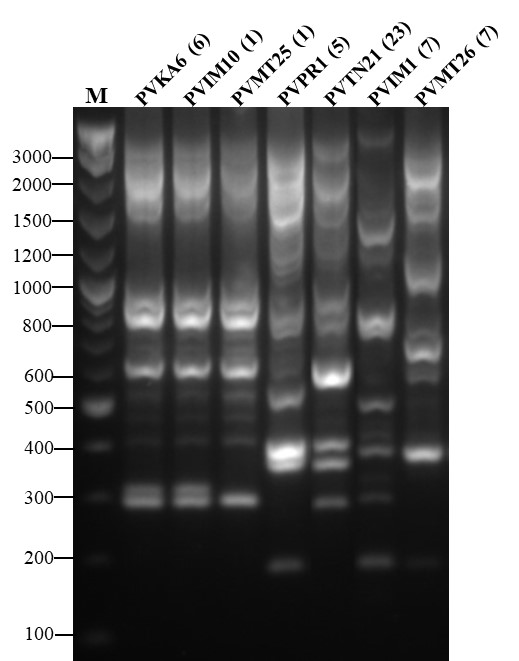


**Supplementary Figure S2.** BOX-PCR fingerprinting patterns of 7 representative rhizobial isolates from field-grown common bean nodules displaying 6 distinct BOX fingerprints. Number of isolates displaying nearly identical profiles were considered as clonal isolates and are indicated within parentheses. Lane M denotes 1 kb DNA ladder and the sizes are indicated in base pairs.


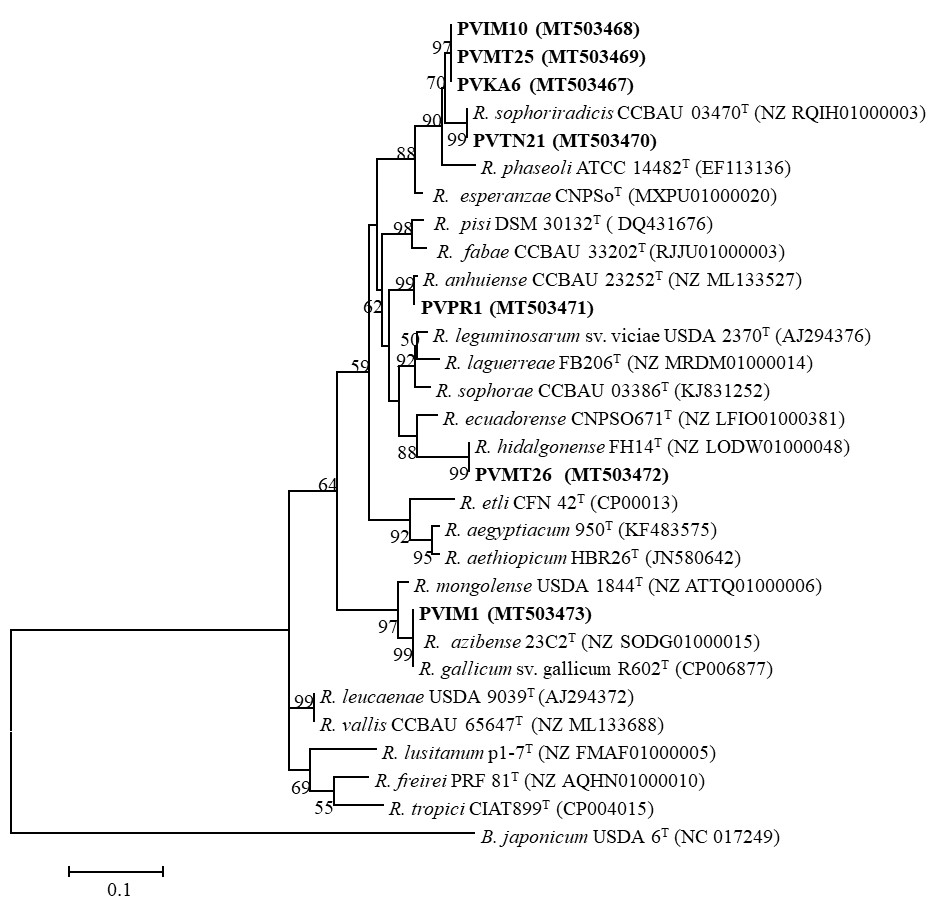


**Supplementary Figure S3.** Maximum likelihood phylogenetic tree based on *recA* gene sequences (462 bp) showing taxonomic relationships of the strains representing different BOX patterns. Strains isolated in the present study are shown in boldface and type strains are indicated by superscript “T”. GenBank accession numbers of the sequences are indicated within parentheses. Bootstrap values (greater than 50%) were calculated for 500 replications and are shown at the nodes. Phylogenetic analysis was conducted in MEGA 6^104^ (<https://www.megasoftware.net/>) using the maximum likelihood algorithm with the Tamura 3-parameter model plus Gamma rate distribution (T92 + G). The scale bar shows the number of nucleotide substitutions per site. *R*., *Rhizobium*.


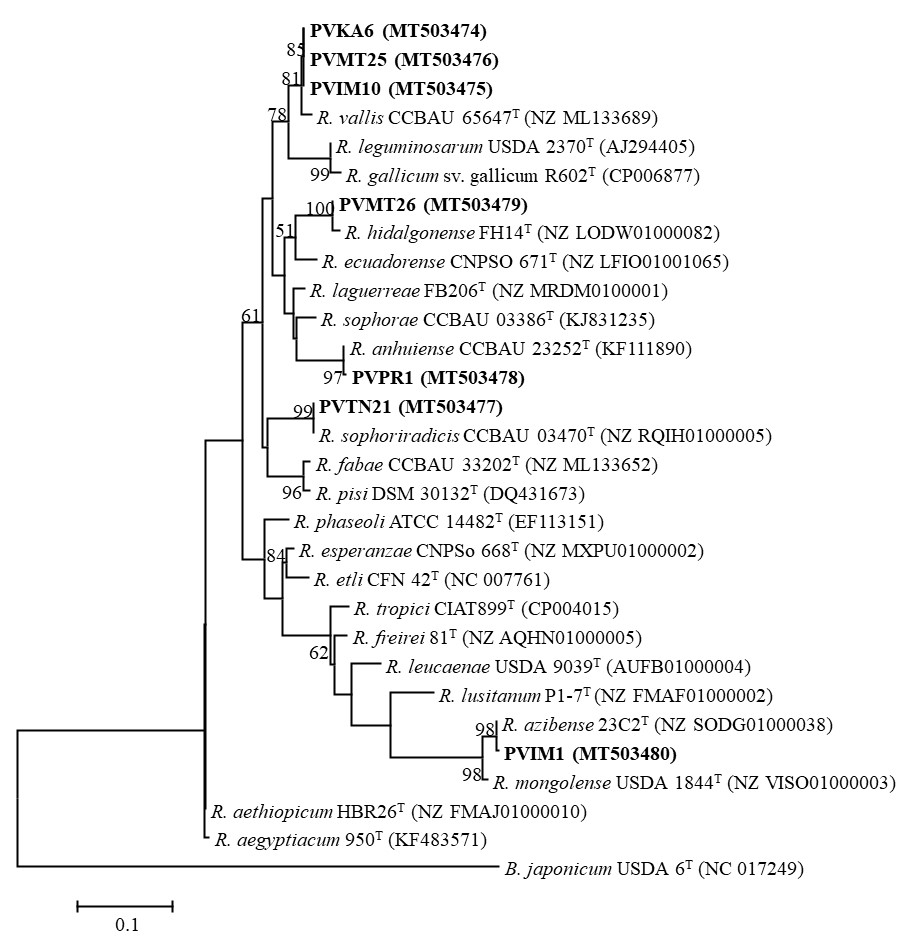


**Supplementary Figure S4.** Maximum likelihood phylogenetic tree based on *atpD* gene sequences (441 bp) showing taxonomic relationships of the strains representing different BOX patterns. Strains isolated in the present study are shown in boldface and type strains are indicated by superscript “T”. GenBank accession numbers of the sequences are indicated within parentheses. Bootstrap values (greater than 50%) were calculated for 500 replications and are shown at the nodes. Phylogenetic analysis was conducted in MEGA 6^104^ (https://www.megasoftware.net/) using the maximum likelihood algorithm with the General Time Reversible model plus Gamma rate distribution (GTR + G). The scale bar shows the number of nucleotide substitutions per site. R., *Rhizobium*.


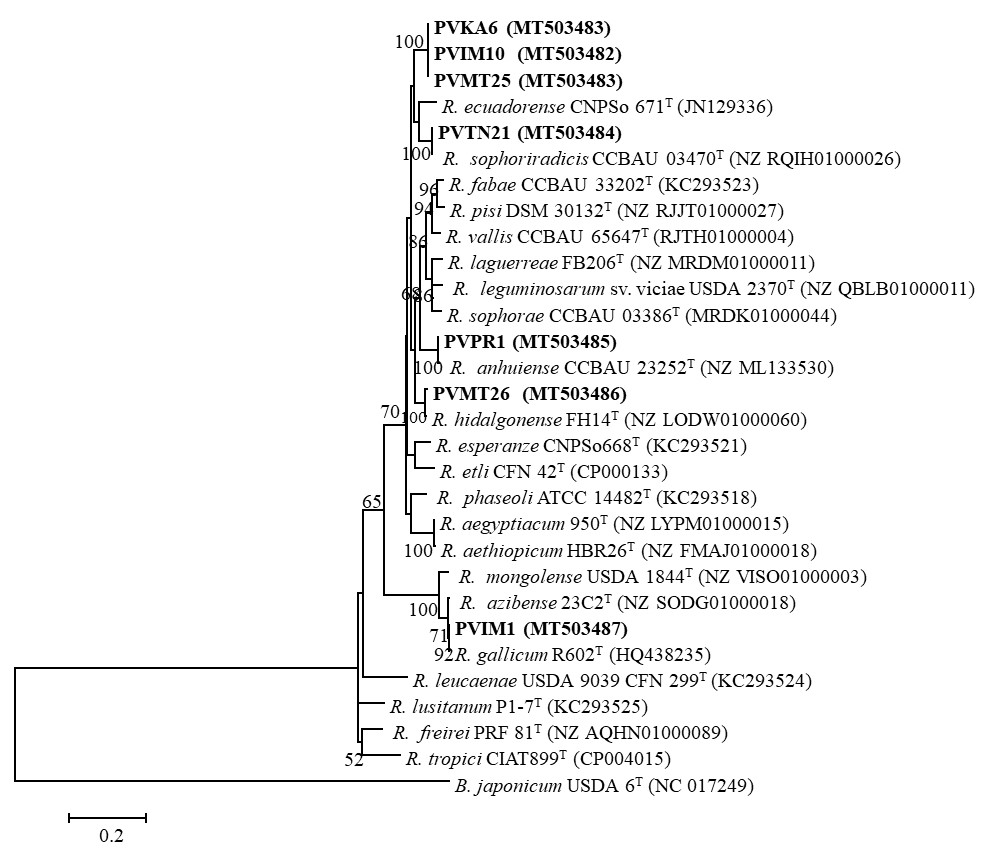


**Supplementary Figure S5.** Maximum likelihood phylogenetic tree based on *gyrB* gene sequences (594 bp) showing taxonomic relationships of the strains representing different BOX patterns. Strains isolated in the present study are shown in boldface and type strains are indicated by superscript “T”. GenBank accession numbers of the sequences are indicated within parentheses. Bootstrap values (greater than 50%) were calculated for 500 replications and are shown at the nodes. Phylogenetic analysis was conducted in MEGA 6^104^ (<https://www.megasoftware.net/>) using the maximum likelihood algorithm with the Tamura 3-parameter model plus Gamma rate distribution (T92 + G). The scale bar shows the number of nucleotide substitutions per site. R., *Rhizobium*.


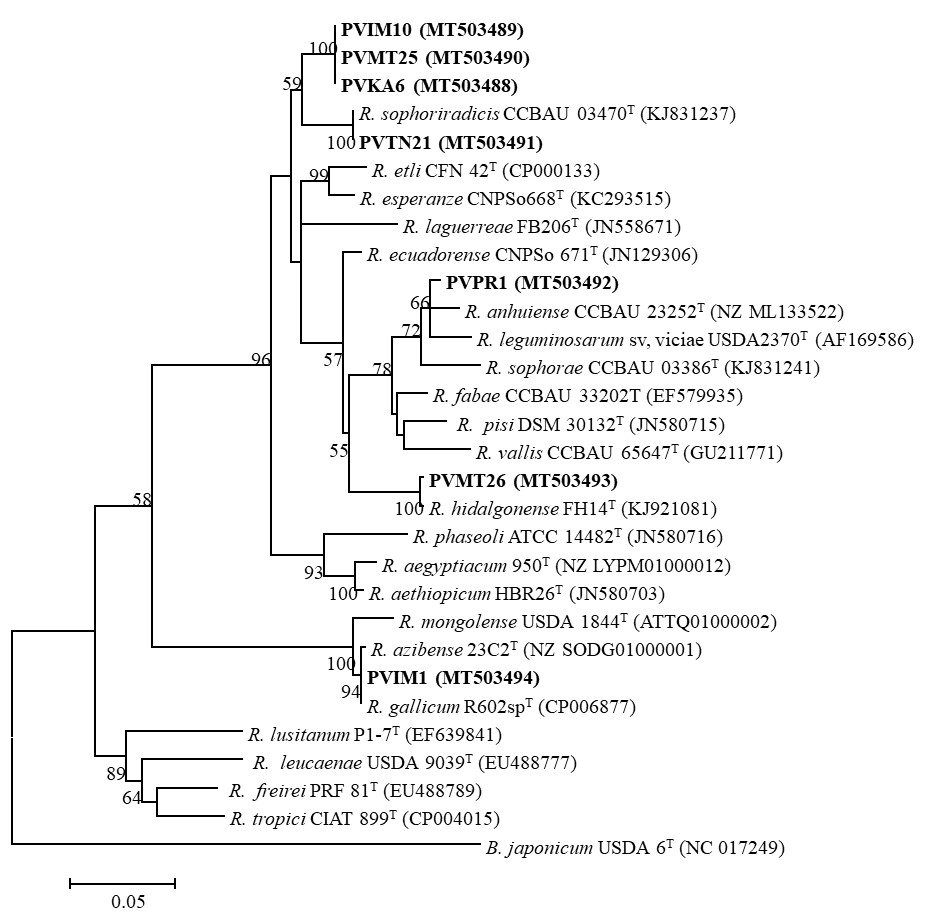


**Supplementary Figure S6.** Maximum likelihood phylogenetic tree based on *glnII* gene sequences (465 bp) showing taxonomic relationships of the strains representing different BOX patterns. Strains isolated in the present study are shown in boldface and type strains are indicated by superscript “T”. GenBank accession numbers of the sequences are indicated within parentheses. Bootstrap values (greater than 50%) were calculated for 500 replications and are shown at the nodes. Phylogenetic analysis was conducted in MEGA 6^104^ (<https://www.megasoftware.net/>) using the maximum likelihood algorithm with the Tamura-Nei model plus Gamma rate distribution plus invariant site (TN93 + G +I). The scale bar shows the number of nucleotide substitutions per site. R., *Rhizobium*.

**
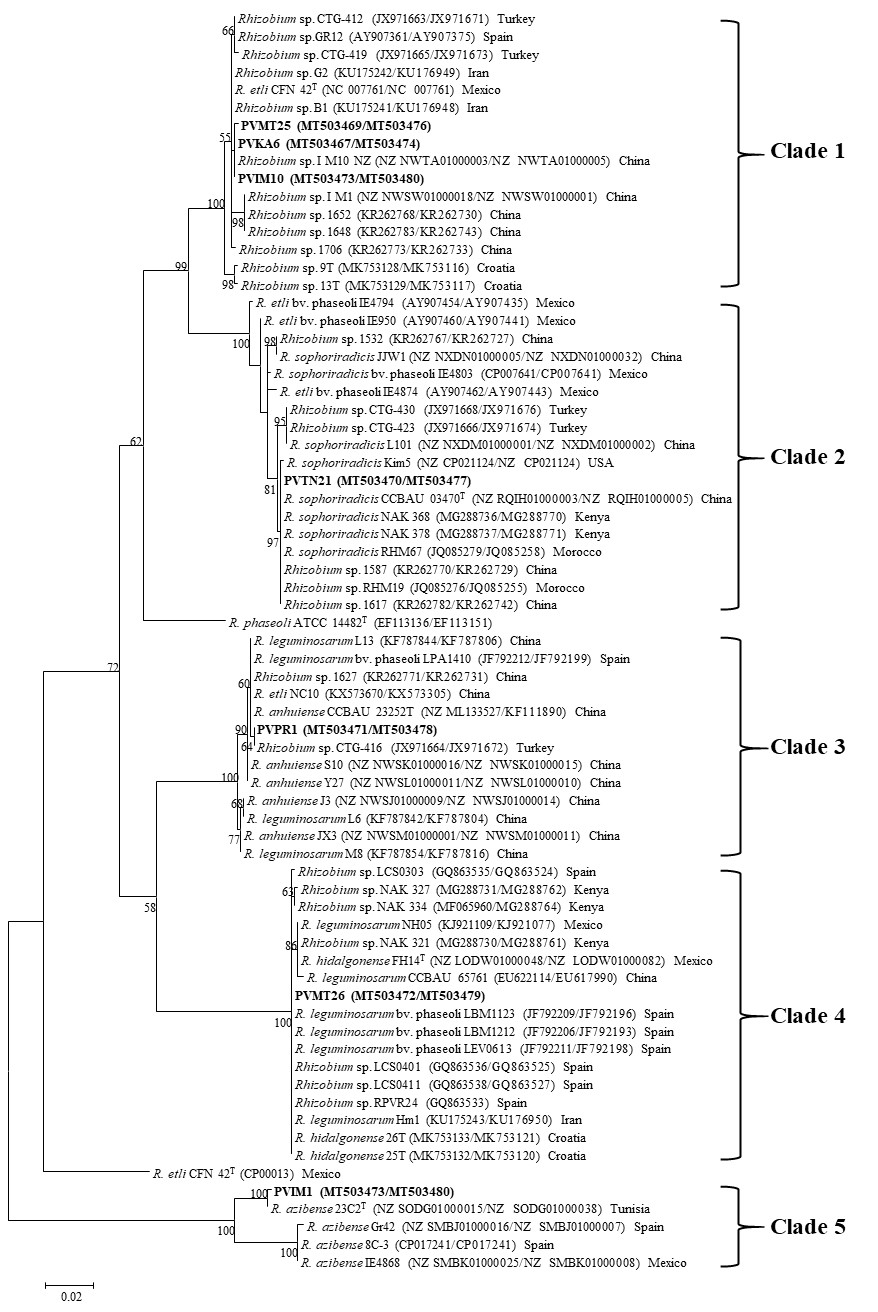
**

**Supplementary Figure S7.** Neighbour-joining phylogenetic tree based on the concatenated *recA*-*atpD* gene sequences (804 bp) showing the phylogenetic relationships between the studied strains and the closest strains isolated in other geographical regions. Strains isolated in the present study are shown in boldface and type strains are indicated by superscript “T”. GenBank accession numbers of the sequences are indicated within parentheses. Bootstrap values (greater than 50%) were calculated for 1000 replications and are shown at the nodes. The scale bar shows the number of nucleotide substitutions per site. Phylogenetic analysis was conducted in MEGA 6^104^ (<https://www.megasoftware.net/>) using the maximum likelihood algorithm with the Tamura Nei-parameter model plus invariant site (TN93 + Ι). *R.*, *Rhizobium*.
